# P-1485. Safety and Immunogenicity of mRNA-1982 and mRNA-1975, mRNA Vaccine Candidates for Prevention of Lyme Disease in Healthy Adults: Interim Results of a Phase 1/2, Randomized, Observer-Blind, Placebo-Controlled, Dose-Ranging Trial

**DOI:** 10.1093/ofid/ofaf695.1670

**Published:** 2026-01-11

**Authors:** Nina H Lin, Christina Dold, Meredith Finn, Jacqueline Dooley, Rujun Teng, Emma Viscidi, Tin Bartholomew, Anthony Rizk, Haixing Wang, Doran Fink

**Affiliations:** Moderna, Inc, Cambridge, MA; Moderna, Inc, Cambridge, MA; Moderna, Inc, Cambridge, MA; Moderna, Inc, Cambridge, MA; Moderna, Inc, Cambridge, MA; Moderna Therapeutics, Cambridge, Massachusetts; Moderna, Inc, Cambridge, MA; Moderna, Inc., Cambridge, Massachusetts; Moderna Inc, Cambridge, Massachusetts; Moderna, Inc, Cambridge, MA

## Abstract

**Background:**

The incidence of Lyme disease, caused by infection with *Borrelia* bacteria, continues to rise globally. Previously developed Lyme disease vaccines based on *Borrelia* OspA antigens elicited antibody responses that correlated with protection; however, no Lyme disease vaccine is currently available for use in humans. Two investigational mRNA vaccines encoding OspA antigens and formulated in a lipid nanoparticle are in development: a monovalent vaccine (mRNA-1982) that covers serotype 1, and a heptavalent vaccine (mRNA-1975) that covers serotypes 1-7.

**Methods:**

This ongoing Phase 1/2, randomized, observer-blind, placebo-controlled dose-ranging trial in healthy adults 18-70 years of age evaluated the safety and immunogenicity of mRNA-1982 and mRNA-1975 at up to 4 dose levels administered as a 3-injection series at 0, 2, and 6 months (NCT05975099). An interim analysis was conducted with follow-up through 28 days after the 3^rd^ injection for safety and reactogenicity evaluation. Immunogenicity was evaluated by serotype-specific anti-OspA IgG binding antibody responses and by levels of LA-2 equivalent antibody (serotype 1 only).

**Results:**

At the interim analysis, 745 of 806 enrolled participants completed the 3-injection study vaccine series. Approximately 1% of enrolled participants were seropositive at baseline for prior *Borrelia* infection. Both mRNA-1982 and mRNA-1975 elicited predominantly mild to moderate reactogenicity that was dose-dependent and increased with each successive injection, with no safety concerns identified. Serotype-specific anti-OspA IgG binding antibody geometric mean-fold rise from baseline and proportions of participants with high levels of LA-2 equivalent antibody responses (≥75% and ≥90% LA-2 inhibition) increased in a dose-dependent manner and with each successive injection across the vaccine groups, compared with no increase in the placebo group.

**Conclusion:**

Interim data from this ongoing Phase 1/2 trial show that both mRNA-1982 and mRNA-1975 are generally safe and elicit dose-dependent reactogenicity and immunogenicity that increased with each of 3 successive injections. These data will inform the product(s), regimen(s) and dose range(s) to be evaluated in future clinical trials.

**Disclosures:**

Nina H. Lin, MD, Moderna, Inc: Stocks/Bonds (Public Company) Christina Dold, PhD, Moderna: Stocks/Bonds (Public Company) Meredith Finn, PhD, Moderna: Stocks/Bonds (Public Company) Jacqueline Dooley, BA, Moderna: Stocks/Bonds (Public Company) Rujun Teng, MS, Moderna Inc.: Advisor/Consultant Emma Viscidi, PhD, MHS, ModernaTX: Employee|ModernaTX: Stocks/Bonds (Public Company) Anthony Rizk, PharmD, MBA, Moderna, Inc.: Employee of Moderna, Inc.|Moderna, Inc.: Stocks/Bonds (Public Company) Haixing Wang, PhD, Moderna, Inc.: Stocks/Bonds (Public Company) Doran Fink, MD, PhD, ModernaTX, Inc.: Employee|ModernaTX, Inc.: Stocks/Bonds (Public Company)

